# Engaging in the Life-planning in Early Alzheimer’s and other Dementias advance care planning intervention is associated with perceived advance care planning concordance and interpersonal connectedness

**DOI:** 10.1093/geront/gnaf179

**Published:** 2025-08-05

**Authors:** Sara G Bybee, Jordana L Clayton, Nancy Aruscavage, Rebecca Utz, Sharon E Bigger, Eli Iacob, Kara Dassel

**Affiliations:** College of Nursing, University of Utah, Salt Lake City, Utah, United States; College of Nursing, University of Utah, Salt Lake City, Utah, United States; College of Nursing, University of Utah, Salt Lake City, Utah, United States; College of Social and Behavioral Science, University of Utah, Salt Lake City, Utah, United States; College of Nursing, University of Utah, Salt Lake City, Utah, United States; College of Nursing, University of Utah, Salt Lake City, Utah, United States; College of Nursing, University of Utah, Salt Lake City, Utah, United States

**Keywords:** Dementia, End-of-life planning, Interpersonal connectedness, Concordance

## Abstract

**Background and Objectives:**

Persons living with dementia often rely upon a care partner as their surrogate medical decision maker, yet little is known about how dementia care dyads achieve advance care planning (ACP) concordance: when a care partner fully understands a care recipient’s values and preferences as best they can. Examining data from a pilot study of the online Life-planning in Early Alzheimer’s and other Dementias (LEAD) intervention to better understand how dyads achieve perceived ACP concordance, we hypothesized that: (1) engaging in ACP was associated with perceived ACP concordance, (2) perceived ACP concordance was associated with interpersonal connectedness, and (3) engaging in ACP was associated with interpersonal connectedness.

**Research Design and Methods:**

Dyads completed the LEAD intervention and answered open-ended survey questions. After aggregating data supporting and or not supporting each hypothesis, process codes and subcodes were used to identify the elements involved in each supported hypothesis (Cohen’s Kappa .65–.82).

**Results:**

*N* = 48 community-based dyads completed the LEAD intervention, with *N* = 43 answering open-ended questions. Care recipients averaged 65.1 years of age (*SD* =14.8); care partners averaged 54.9 years (*SD* = 14.6) and were primarily spouses (*n* = 32, 66.7%) or children (*n* = 12, 25.0%). Engaging in ACP was associated with higher interpersonal connectedness and with perceived ACP concordance. Perceived ACP concordance was not associated with higher interpersonal connectedness.

**Discussion and Implications:**

These findings suggest that clinicians should focus on facilitating ACP discussions with dyads, as these conversations appear crucial for fostering understanding and agreement between dyad members, ultimately leading to perceived ACP concordance.

As of 2023, roughly 6.9 million Americans aged 65 and older were living with Alzheimer’s disease and related dementias (ADRD) (Rajan et al., 2021). As the population ages, the incidence of dementia will likely increase, as the risk of dementia increases with age (Alzheimer’s Association, 2023). Dementia, (hereinafter used to refer to ADRD), is a unique chronic illness in that as the disease progresses, the person living with dementia is likely to lose their decision-making capacity. Advance care planning (ACP), “A process that supports adults at any age or stage of health in understanding and sharing their personal values, life goals, and preferences regarding future medical care,” is one way to ensure that the end-of-life values and preferences of persons living with dementia are followed (Sudore et al., 2017, p. 670). Research illustrates that individuals who engage in ACP demonstrate better physical and mental health outcomes, as do their care partners—ACP has been shown to reduce unwanted medical procedures and hospitalizations and improve the quality of life (Dixon et al., 2018).

Advance care planning often involves identifying a surrogate medical decision-maker who will act on behalf of a person living with dementia once they lose decision-making capabilities. When surrogate decision makers feel unprepared to make medical decisions on behalf of a care recipient, they can experience depression, anxiety, and even trauma-related symptoms (Anderson et al., 2009; Azoulay et al., 2005). Taken together, these findings suggest that preparing surrogate medical decision-makers to act on behalf of persons living with dementia can result in improved outcomes for both members of the dyad, such as an improvement in dyadic concordance on end-of-life care, care partners’ confidence in surrogate decision-making, and the quality of end-of-life communication (Liu et al., 2024). In our previous work, we defined concordance as, “A mutual understanding with the dyad of the care recipient’s ACP values and preferences, and a commitment by the surrogate decision maker to carry out these values and preferences to the best of their ability” (Clayton et al., 2024a, p. 1).

The Life-planning in Early Alzheimer’s and other Dementias (LEAD) Guide is a dementia-specific ACP guide developed by our team. It contains questions related to the end-of-life values and preferences of persons living with dementia such as their preferred location of care and death, decisions regarding medical interventions and resuscitation, and preferences surrounding controlling the timing of their death (Dassel et al., 2019). The LEAD Guide not only helps a care recipient plan for their long-term and end-of-life care but also helps prepare a care partner for their future role as a surrogate decision-maker. Developed with input from dementia care dyads and dementia experts, the LEAD Guide informed the development of the LEAD intervention.

The LEAD intervention was developed through the research team’s partnership with a Community Advisory Board (CAB) (Clayton et al., 2024b). In the online intervention, dementia care dyads completed three modules that guided them through the completion of the LEAD Guide and facilitated a discussion of their responses in an attempt to improve ACP concordance and prepare surrogate decision makers. As few dementia-specific dyadic ACP interventions exist, we sought to (1) test the initial efficacy and feasibility of this intervention (reported in Iacob et al., 2025, under review) and (2) gain a greater depth of understanding regarding how dyads achieve ACP concordance—that is, the *process* of achieving concordance rather than the outcome. To fill the gap in knowledge regarding how dyadic ACP interventions in dementia can foster care dyad ACP concordance, this study analyzed participants’ open-ended survey responses. Informed by prior research, we hypothesized that data from dyads who completed the LEAD intervention (those who engaged in ACP) would illustrate perceived dyadic ACP concordance and interpersonal connectedness and that perceived ACP concordance alone would also be associated with high interpersonal connectedness.

## Method

### Conceptual model

**Figure 1.**
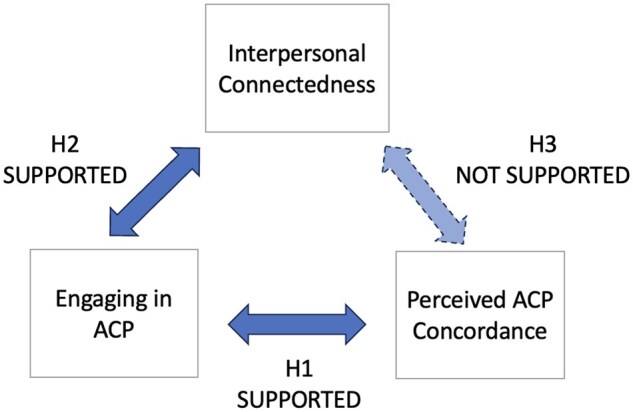
Hypothesized conceptual model. H1 = hypothesis 1; H2 = hypothesis 2; H3 = hypothesis 3; ACP = advance care planning.

This study was informed by prior research demonstrating associations between the completion of advance directives and improved individual and dyadic outcomes for care recipients and care partners facing dementia. Previous studies demonstrate that completing advance directives can help prepare surrogate decision makers—which increases their confidence in surrogate decision-making and helps avoid increased risk of depression, anxiety, and trauma symptoms—improve end-of-life communication and foster dyad concordant end-of-life care (Anderson et al., 2009; Azoulay et al., 2005; Dixon et al., 2018; Liu et al., 2024). We hypothesized that engaging in ACP via the LEAD intervention would be associated with higher interpersonal connectedness and perceived ACP concordance (see Figure 1 for a representation of our hypothesized conceptual model). Informed by our conceptual model, our hypotheses were:

H1: Engaging in ACP is associated with perceived ACP concordance.

H2: Engaging in ACP is associated with higher interpersonal connectedness.

H3: Perceived ACP concordance is associated with higher interpersonal connectedness.

In seeking to understand the lived experiences of dementia care dyads engaging in ACP, we took an interpretive phenomenological analysis approach, which aims to gain a detailed understanding of a research participant’s perspective (Smith et al., 1999). Phenomenological analysis can be especially useful in healthcare contexts, as it can provide insight into the care recipient’s or end-user’s experience (Biggerstaff & Thompson, 2008).

### Setting and sample

This study was approved by the University of Utah IRB #00132042. Participants were recruited through Research Match (Harris et al., 2012) and TrialMatch (Alzheimer’s Association, n.d.), two national research registries developed by the National Institutes of Health and the Alzheimer’s Association. Also, CAB members assisted in identifying potential participants. Interested participants received an email via the electronic survey platform Qualtrics^XM^ (Qualtrics, Provo, Utah), including informed consent and baseline assessments.

Participants included community-dwelling persons at risk of or living with dementia and their care partners. Eligible at-risk dyads were those in which one member self-reported they were at risk of or experiencing perceived memory loss. The other member of the dyad was required to be a designated care partner. Care partners were required to be 18 years or older; care recipients fifty years or older, and both dyad members were required to be able to complete online questionnaires and all study procedures. For additional information on inclusion and exclusion criteria, see Iacob et al. (2025; under review).

### Data collection

Participants completed baseline surveys via Qualtrics software. The following demographic information was collected at baseline: sex, age, race, ethnicity, marital status, highest level of education completed, employment status, household income, religiosity/spirituality, and religion. In addition, three questions regarding computer and internet use were asked to assess individuals’ computer literacy.

Participants then completed the LEAD intervention. In ­Module 1, the care recipient completed the LEAD Guide (Dassel et al., 2019) via the online platform and the care partner completed the LEAD Guide as if they were the care recipient. Activities in Module 2 instructed the participant living with dementia and their care partner to have a discussion regarding each of their responses to the LEAD Guide, focusing on any questions in which their responses differed. For example, if the care recipient indicated that they would want to live as long as possible even if their quality of life was poor, but the care partner thought the person with dementia would want to have a higher quality of life, even if that meant it was shorter in duration, then the care dyad was instructed to discuss these different responses to the LEAD guide questions between themselves. Finally, Module 3 had care recipients revise their LEAD guide as necessary, complete their state’s advance directive document, and share their completed LEAD Guide and advance directive with family members, friends, and their medical care team. (See Table 1 for a timeline of intervention procedures and post-intervention surveys.) After completing all three modules, participants received postintervention surveys at 12, 24, and 36 weeks that asked about dyad members’ relationship quality, decision-making self-efficacy, and experiences with the LEAD intervention.

**Table 1.** Timeline of intervention components.

Intervention components	Baseline Week 1	Module 1 Week 4	Module 2 Week 7	Module 3 Week 10	Post-I Week 12	Post-I Week 24	Post-I Week 36
Procedures	1. Watch video 1 2. Consent 3. Complete demographics	1. Watch video 2 2. Complete LEAD Guide 3. Complete assessments	1. Watch video 3 3. Discuss completed LEAD Guide 5. Complete assessments	1. Watch video 4 2. Complete LEAD Guide 3. Complete assessments	1. Complete assessments	1. Complete assessments	1. Complete assessments
Video content	Study overview and informed consent	Module instructions and importance of ACP	Module instructions and conversation fundamentals	Module instructions and process of ACP dissemination			
LEAD Guide		X-care recipient only		X-care recipient only			
Perceived ACP congruence		X-care partner only	Open-text box		Open-text box	Open-text box	Open-text box
Video recording			X				
Acceptability, usability, and feasibility		X	X	X	X	X	X
		Open text box	Open text box	Open text box	Open text box	Open text box	Open text box

*Note.* Care-recipients and care partners fill out each assessment individually unless specified otherwise. *Feasibility*: enrollment and retention; *Usability*: ease of use of intervention items; *Acceptability*: satisfaction with intervention items. LEAD = Life-planning in Early Alzheimer’s and Dementia; ACP = advance care planning.

### Data analysis

Demographic data and closed-ended survey data were analyzed using SPSS version 28.0 (SPSS Statistics, Chicago, IL) (IBM, 2018). Descriptive statistics included means, standard deviations, frequencies, and percentages as appropriate. Comparisons of care recipient and care partner demographics were conducted with Student’s *t*-tests and chi-square tests as appropriate. For qualitative analysis of the open-text responses (which were obtained independently from each dyad member throughout the intervention and postintervention period), all data were exported from Qualtrics into Excel spreadsheets and then into portable document format (PDFs) so that each PDF contained the open-text responses from one dyad. The PDFs were imported into NVivo qualitative analysis software (QSR, 2018). Two coders (S.G.B. and J.C.) read through all open-text responses to gain familiarity with the content. To enhance the trustworthiness and reflexivity of our findings, the coders used memos to note initial observations and routinely discussed these observations amongst themselves and with the entire research team. We utilized process codes in our first round of coding as processes, “imply actions intertwined with the dynamics of time, such as things that emerge, change, occur in particular sequences” (Miles et al., 2020, p. 66). First round process codes included: achieving ACP concordance, engaging in ACP, and fostering higher interpersonal connectedness.

After identifying process codes, we determined the extent of support for our hypotheses by developing a matrix in which each dyad’s responses were listed in one row and related data were placed in each column that supported or did not support the hypothesis in question (see Table 2 for an example of how this matrix was designed). By placing one dyad member’s comments next to the comments of the other dyad member, we were able to conduct dyadic qualitative analyses which “better understand and identify overlaps and contrasts between members of the couple” (Collaço et al., 2021, p. 1558). Coded data from each dyad was reviewed and placed within the appropriate column if it supported hypotheses 1, 2, or 3, or if it refuted hypotheses 1, 2, or 3 (Miles et al., 2020). To further understand the process by which care partners and care recipients achieved dyad ACP concordance, we then conducted second round coding using the matrix containing first round codes to identify subcodes within each process code. Using the list of process codes and subcodes, a codebook containing code and subcode labels, their definition, and example quotations was drafted by S.G. B. The codebook was reviewed by J.C. and changes were made to definitions and example quotations until both coders agreed on all codebook content (see online Supplementary Material for the qualitative codebook).

**Table 2.** Example matrix of data supporting and not supporting hypotheses.

Dyad No.	**Support for H1** Engaging in ACP is associated with achieving perceived ACP concordance	**Support for H2** Engaging in ACP is associated with fostering higher interpersonal connectedness.	**Support for H3** Achieving perceived ACP concordance is associated with fostering higher interpersonal connectedness.	**Not supportive of H1** Engaging in ACP is *not* associated with achieving perceived ACP concordance.	**Not supportive of H2** Engaging in ACP is *not* associated with fostering higher interpersonal connectedness.	**Not supportive of H3** Achieving perceived ACP concordance is *not* associated with fostering higher interpersonal connectedness
001	“Confident that [care partner] will carry out my wishes as discussed and agreed upon.” “I understand [care recipient’s] end of life wishes. They are documented in his will.” “I can be counted on to make the hard decisions when its time”	“Conversation was easy and comfortable…we discussed most of the issues recently as we prepared our wills.”	“We are closer in mind and spirit. We express our love for each other more.”	-	“We didn’t have any disagreements. However, I didn’t think I let him know how frustrating it is to deal with his diagnosis and how hard it is on me when he took so long to exercise.”	“We have grown closer in order to deal with medical issues that affect both of us.”
016	“She understands.”	-	-	“Conflicts over her abuses of pain medications/treatments.” “She remains difficult to monitor 100% of time.”	“Overall better since drinking curtailed x 1 month.” “He has had a stroke and has a tumor in his brain. He finally agreed to go the ER. I must say I got impatient, as these symptoms can be a sign of a brain bleed. This time he had no brain bleed. We will not wait this long if it happens again.”	-

*Note.* H1 = hypothesis 1; H2 = hypothesis 2; H3 = hypothesis 3; ACP = advance care planning.

Both coders utilized this revised codebook to double-code 10% of the data (open-text responses from five dyads). The following process of unitization was used to improve intercoder reliability: (1) The primary coder coded the transcript using the codebook, (2) the primary coder removed the code labels from the coded excerpts but left the excerpts highlighted, and (3) the second coder applied code(s) to each highlighted excerpt using the codebook (Campbell et al., 2013). This process improves intercoder reliability by removing the need for the second coder to establish the length of a code in addition to the application of the code name (Campbell et al., 2013). Intercoder reliability was calculated using NVivo software, resulting in a Cohen’s Kappa of .82, indicating almost perfect agreement (Landis & Koch, 1977). The two coders met and discussed coding discrepancies until consensus was reached. Both coders revised the coding rules and code definitions until consensus was reached on all codebook material.

At this point in time, a third coder and content expert (K.D.) was brought onto the team and introduced to the codebook to ensure greater reliability and to validate codes and subcodes. All three coders coded an additional 10% of the data (five additional transcripts). Again, intercoder reliability was calculated using NVivo software, resulting in a Cohen’s Kappa of .65, or substantial agreement (Landis & Koch, 1977). The codebook was revised once more after all coders met to discuss discrepancies. The remaining open-text responses were then divided amongst all coders. Those conducting qualitative coding included two PhD prepared scientists, with extensive experience conducting health and social science research with older adults, and one graduate research assistant who was completing a master’s degree in economics and had worked with this research team for many years prior to engaging in this analysis.

## Results

### Demographic characteristics

A total of 48 dyads completed all study procedures. Of these participants, 43 dyads (*N* = 86 participants) completed open-ended questions and were included in this qualitative analysis. Care recipients were older with a mean age of 65.1 (*SD* = 14.8, *R* = 28-89) compared to care partners’ whose mean age was 54.9 (*SD* = 14.6, *R* = 29-84; *t*(93) = 3.38, *p* = .0011). Care partners were primarily spouses or long-term partners of the care recipient (*n* = 32, 66.7%) or children (*n* = 12, 25.0%). Roughly half of care recipients (*n* = 25, 52.1%) were female and reported concerns about cognitive decline including family history or experiencing changes in memory (*n* = 26, 54.2%), with 17 (35.4%) having a formal diagnosis of mild cognitive impairment (MCI) or dementia (see Table 3 for additional demographic characteristics).

**Table 3.** Demographic characteristics.

Variables	Subgroup	Number of care recipients (*n* = 48)	Percent of care recipients	Number of care partners (*n* = 48)	Percent of care partners
		*n*	%	*n*	%
Age	*M* (*SD*)	65.1	14.8	54.9	14.6
	Median (range)	67	(28, 89)	56	(29, 84)
	Missing	1			
Relationship to care recipient	Parent	–	–	1	2.1
	Child	–	–	12	25
	Spouse/long-term partner	–	–	32	66.7
	Other	–	–	3	6.3
Current situation	Concern, family hx, changes	26	54.2	–	–
	MCI or dementia diagnosis	17	35.4	–	–
	Missing	5	10.4	–	–
Diagnosis	Alzheimer’s disease	7	14.6	–	–
	Lewy body dementia	1	2.1	–	–
	Frontotemporal dementia	1	2.1	–	–
	Mild cognitive impairment	7	14.6	–	–
	Other	1	2.1	–	–
	No diagnosis	31	64.6	48	100
Gender	Male	21	43.8	12	25
	Female	25	52.1	35	72.9
	Missing	2	4.2	1	2.1
Race	Asian	2	4.2	2	4.2
	Native Hawaiian/Pacific Islander	4	8.3	4	8.3
	White	39	81.3	41	85.4
	Multiracial	1	2.1	1	2.1
	Black or African American	1	2.1	0	0
	Missing	1	2.1	0	0
Ethnicity	Not Hispanic	43	89.6	45	93.8
	Hispanic	4	8.3	2	4.2
	Missing	1	2.1	1	2.1
Marital status	Married/living with a partner	36	75	44	91.7
	Divorced	4	8.3	1	2.1
	Widowed	6	12.5	0	0
	Single	1	2.1	3	6.3
	Missing	1	2.1	0	0
Education	High school graduate or GED	4	8.3	2	4.2
	Some college, Assoc, other	10	20.8	7	14.6
	College degree	19	39.6	25	52.1
	Postgraduate	14	29.2	14	29.2
	Missing	1	2.1	0	0
Employment	Full-time	7	14.6	15	31.3
	Part-time	4	8.3	8	16.7
	Not employed	11	22.9	5	10.4
	Retired	25	52.1	20	41.7
	Missing	1	2.1	0	0

*Note. SD* = standard deviation; hx = history; MCI = mild cognitive impairment; GED = general education development test; Assoc = associate’s degree.

### Qualitative results

#### Process codes

We found that two of our hypotheses were supported by the data from dyads’ open-text responses; Hypothesis 1 that engaging in ACP is associated with perceived ACP concordance was supported by data from 29 dyads. Hypothesis 2 that engaging in ACP is associated with higher interpersonal connectedness was supported by data from 10 dyads. Hypothesis 3 that perceived ACP concordance is associated with higher interpersonal connectedness was not supported (there were data from one dyad that supported this hypothesis and data from one dyad that did not support this hypothesis) (Table 2).

#### Hypothesis 1 subcodes (engaging in ACP is associated with perceived ACP concordance)

We identified three subcodes within the data that supported hypothesis 1: discussion and clarification, understanding and agreement, and confidence (see Table 1 in online Supplementary Material for the codebook and see Table 4 for an example of how subcodes identified within hypothesis 1 process codes). Data largely demonstrated that discussion and clarification led to understanding and agreement, which then fostered confidence. For example, one care recipient stated, “Confident that [care partner] will carry out my wishes as discussed and agreed upon.” Another care recipient noted, “We had a few areas that we differed in [the responses] ‘agree’ and ‘strongly agree’ but since we have had discussions we were in agreement in my wishes.” Similarly, a care partner explained, “I have a high level of confidence in my ability to make decisions that reflect his end-of-life wishes… since I have had these conversations with him I know I will be able to make decisions with confidence.” These quotations demonstrate the relationship between engaging in dialogue about ACP and a mutual understanding of the care recipient’s values and preferences—both of which then foster confidence in the care recipient that these will be followed and confidence in the care partner that they know what the care recipient wants and will be able to follow through with those values and preferences.

**Table 4.**
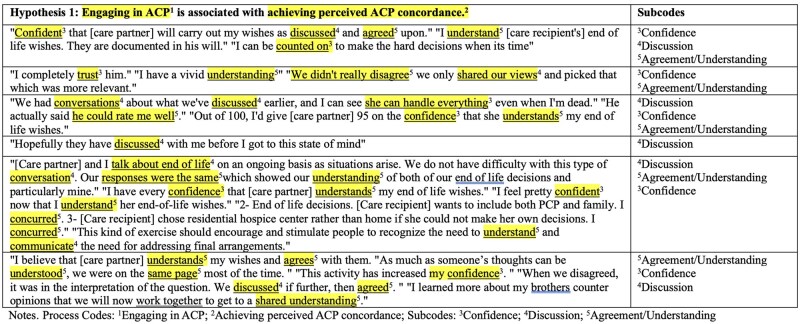
Subcodes identified within Hypothesis 1 process codes. ACP = advance care planning; Process Codes: ^1^Engaging in ACP; ^2^Achieving perceived ACP concordance; Subcodes: ^3^Confidence; ^4^Discussion; ^5^Agreement/Understanding.

#### Hypothesis 2 subcodes (engaging in ACP is associated with higher interpersonal connectedness)

We identified three subcodes within the data supporting H2, that engaging in ACP is associated with fostering higher interpersonal connectedness. These subcodes were named ease of discussion, unity, and transparency. When dyads spoke of their conversation about the LEAD Guide, many noted that the conversation was easy or comfortable. Those that made these observations about their discussion also explained this ease and comfort because of ACP discussions held prior to the LEAD intervention. For example, one care recipient stated, “It was comfortable and easy for me, partly because my father has Alzheimer’s, so I’ve already been talking to my family in very straight-forward ways about my end-of-life wishes.” Similarly, a care partner explained that their conversation about the LEAD guide “was easy and comfortable… we discussed most of the issues recently as we prepared our wills.”

The second subcode within data supporting H2 was transparency. This involved dyads expressing their feelings and their end-of-life values and preferences. Dyads described appreciating engaging in an open conversation: “I can talk open[ly] about just about anything. Feel accepted and safe. He felt heard and glad we spoke about these topics. Nice to hear him express his point of view on this studies content.” Another care recipient remarked, “Although we were very close in our answers it gave us a chance to discuss our feelings and to better understand the reasoning thoughts. Although I have shared my wishes to my son and family it was good to discuss the areas that my son was not aware of my medical issues.” Transparency also involved one dyad member communicating personal experiences that the other dyad member may not have known: “We had a long discussion about involving a palliative care doctor in my team. He was not aware of how that works, and he was not aware of how important that person was when my best friend died. I feel better knowing that he understands that he would not have to make this decision.”

The final subcode, unity, involved feelings of closeness, spending more time together, and feeling like a team. As described earlier, engaging in ACP led to transparency, which was key in fostering better interpersonal connectedness: “Because each effort to re-visit the subject is based upon an evident need for me to provide more assistance and partnership, [care recipient] is growing in her reassurance that I am standing with her, not over her, and that we can successfully face all of this together”; “We did work through feelings and being heard. Yes it was resolved and it felt good.”

#### Data not supporting hypotheses

Examining the columns containing data that did not support the hypotheses, we found that the data from four dyads did not support H1. The data from these dyads spoke to conflicts related to drug abuse and burdening the care partner, as well as the timing of the LEAD intervention. For example, a care partner said, “This exercise would have been more beneficial prior to his cognitive decline.” Data from six dyads did not support H2. Of these dyads, five specifically described strained relationships due to the care recipients’ disease or declining health. For example, a care provider reported that the care recipient, “continues to decline and has become incontinent which causes many accidents/clean ups and laundry. He refuses to wear disposable underwear and this is a significant source of conflict.”

Data from one dyad supported H3 while data from another dyad did not. Thus, we concluded that H3 was not supported. A dyad stated that their closeness developed as a result of their experiences with both dyad members’ medical issues, suggesting that interpersonal connectedness was not necessarily fostered by achieving perceived ACP concordance, but by shared medical concerns. Alternatively, one dyad described how achieving perceived ACP concordance has led to the understanding that both dyad members will need to work together and in order to engage in meaningful ACP. The care partner stated, “As much as someone’s thoughts can be understood, we were on the same page most of the time” and “I learned more about my brothers counter opinions that we will now work together to get to a shared understanding.” These comments may illustrate that perceived ACP concordance fostered interpersonal connectedness.

Data from one additional dyad did not support any of our hypotheses. For this dyad, while the care recipient reported that the care partner “definitely understands” the care recipient’s end-of-life wishes, the “question is about accepting my wishes and carrying them out.” The care partner felt that, “One place we certainly disagreed is that she would like to live with me and completely be taken care of by me for the rest of her life and I told her that I would rather have her in a separate house close to me or in a nursing home or something like that. Other than that we disagree on how much she wants me to take care of the rest of our family members I do not want the responsibility but she is strongly battling against me on that. It’s impossible for me to [accept] that my grandmother is asking me to take care of these two people after she’s gone.” In this instance, engaging in ACP did not assist in achieving perceived ACP concordance with the exception of the care partner’s understanding of the care recipient’s wishes. In this case, engaging in perceived ACP was not associated with a higher interpersonal connectedness and instead, was associated with conflict between the care partner and her grandmother, the care recipient.

## Discussion

The fact that our study discovered that engaging in ACP was associated with achieving perceived ACP concordance aligns with previous research demonstrating that engaging in ACP can inform goal concordant end-of-life care (Liu et al., 2024). Our study adds new insight into the process by which dementia care dyads achieve perceived ACP. Our findings demonstrate that an intervention facilitating discussion and clarification led to understanding and agreement, which then fostered confidence in both dyad members—the confidence of the surrogate decision-maker to follow through with the care recipients’ end-of-life values and preferences and the confidence of the care recipient that their instructions would be followed. A 2025 study of persons with end-stage heart failure similarly identified that ACP served as a “catalyst for crucial conversations” (Róin et al., 2025, p. 405), “fostered a sense of clarity” (p. 407), and helped them “maximize meaningful moments with their loved ones” (p. 407).

This study also provides additional insight into the components integral in the association between engaging in ACP and better interpersonal connectedness: ease of discussion, unity, and transparency. Our findings counter prior qualitative research which reported that care partners were unable to discuss ACP with the person living with dementia as they worried it could cause this person to feel as if death were imminent (Sellars et al., 2019). These findings are also important in light of recent concerns that ACP does not result in goal-concordant end-of-life care (Morrison et al., 2021).

While a minority of participants reported strained relationships, difficulty in discussing ACP, and or divisiveness and discordance in their dyads, these dyads also connected this dissonance to the care recipients’ decline or to preexisting and ongoing relationship strain. Our finding mirrors recent research examining caregiving for older adults with dementia, which found that historical relationship dynamics inform future interactions and discussions between the person living with dementia and their care partner (O’Conor et al., 2025). In addition to discovering that prior relationship dynamics play into current discussion dynamics, the aforementioned study also aligns with our findings in its description of the struggle involved in retaining patient autonomy while also recognizing the limitations of the person living with dementia due to cognitive decline (O’Conor et al., 2025). Ultimately, our findings suggest that the LEAD intervention is promising in fostering discussion, clarification, agreement, understanding, and ultimately, confidence in the future surrogate decision maker to carry out the care recipient’s end-of-life preferences. Furthermore, our results demonstrate that unity between dyad members, ease of discussion, and transparency during conversation about ACP is associated with better interpersonal connectedness.

These findings may have important clinical implications for many health professionals. For instance, our findings support the need for ACP to encompass more than the mere completion of an advance directive or document such as a Physician Order for Life Sustaining Treatment or Medical Orders for Life Sustaining Treatment. As discussion/clarification is critical in fostering agreement/understanding, which then fosters confidence—the care recipient’s confidence in their care partner’s ability to follow through with their end-of-life preferences, and the care partner’s confidence in their ability to serve as a surrogate decision maker—discussion and understanding may be necessary aspects of ACP. These findings align with previous research which demonstrated that among those aged 85 and older, 28.4% added new limitations to orders regarding their life sustaining treatment, compared with 3.2% of those younger than 65 years of age (Kim et al., 2016). Thus, because advance directives can change, especially among older adults who are at greater likelihood of developing dementia, ongoing and repeated conversations about ACP are needed. Without discussion and understanding between dementia care dyad members, confidence may be more difficult to achieve, which, as noted earlier, can negatively affect both dyad members’ wellbeing (Anderson et al., 2009; Azoulay et al., 2005).

Our findings may also suggest that when dyads exhibit more ease in ACP discussions, transparency, and unity around ACP and end-of-life decisions, this may reflect a strong relationship and could indicate that the dyad is not in need of additional guidance surrounding ACP. Alternatively, if a dyad seems to have a great deal of difficulty having discussions about ACP and seems divided or one dyad member seems disengaged or withdrawn, this may signal a dyad in need of additional guidance. Other dyads in need of additional support and guidance may include those who are further along in the dementia trajectory, who have historically strained relationships, or who are often in conflict with one another. Literature shows that even in individualistic societies such as Switzerland, having complete trust in one’s relatives is associated with higher engagement in ACP, findings which align with our results (Iunius et al., 2024).

### Limitations

While our qualitative results may suggest that the LEAD intervention was associated with better interpersonal connectedness, our findings may also reflect a selection bias in that dyads who agreed to participate in this study may have had higher interpersonal connectedness at baseline and have felt more comfortable discussing end-of-life values and preferences (as they agreed to do so for this study). As we analyzed open-ended responses throughout the entire study, including the final follow-up questionnaires, we were unable to engage in member checking. Additionally, given the cross-sectional nature of the data, we cannot claim causality in the relationships between engaging in the LEAD intervention and higher interpersonal connectedness. The data from this pilot study were generated from participants both with and without formal diagnoses of cognitive decline, and information on disease stage and cognitive status was not collected. Thus, it is possible that a sample composed entirely of individuals with formal diagnoses and their care partners could result in differences regarding the LEAD intervention, particularly among those in the later stages of the disease who are more likely to have difficulty engaging in the intervention. In addition, our sample was primarily non-Hispanic White and highly educated, and therefore our findings may not be generalizable beyond similar groups. Finally, as this was a pilot study, we did not test the LEAD intervention’s effectiveness in achieving perceived ACP concordance against other ACP interventions. Despite these potential limitations, our findings contribute a greater depth of knowledge into the processes involved in achieving perceived ACP among dementia care dyads.

## Conclusion

For community dwelling dementia care dyads, achieving perceived ACP concordance is especially critical, as care partners become increasingly relied upon to act as the care recipient’s medical surrogate decision-maker. Although research suggests that ACP planning can be beneficial for care recipients and care partners, there is little understanding, particularly among dementia care dyads, regarding how dyads reach perceived ACP concordance, and its association with the dyad’s interpersonal connectedness. This pilot study adds new knowledge to the literature by illuminating the associations between interpersonal connectedness, perceived ACP concordance, and engagement in ACP. Our current NIH Stage IB clinical trial (R01AG069033) expands upon this knowledge by testing the online LEAD intervention with a larger sample of dementia care dyads with subjective cognitive complaints or a diagnosis of MCI or dementia. With projections that the number of Americans living with Alzheimer’s disease will double by the year 2050, understanding how to foster perceived ACP concordance and positive outcomes is more crucial than ever (Hebert et al., 2001).

## Supplementary Material

gnaf179_Supplementary_Data

## Data Availability

The data used in this manuscript are available upon request due to privacy concerns. This study was not preregistered.
