# Supplementary material for: Engaging in the Life-planning in Early Alzheimer’s and other Dementias advance care planning intervention is associated with perceived advance care planning concordance and interpersonal connectedness
Source: Gerontologist. 2025 Aug 5;65(10):gnaf179. doi: 10.1093/geront/gnaf179 (PMC12494457; doi:10.1093/geront/gnaf179)
Supplement: gnaf179_Supplementary_Data [file gnaf179_supplementary_data.docx]

Supplemental Material

Table 1. Codebook

| **Hypothesis** | **Process Codes** | **Subcode** | **Definition** | **Example quotation** |
| --- | --- | --- | --- | --- |
| H1: Engaging in ACP is associated with perceived ACP concordance. | Engaging in ACP  Achieving perceived ACP concordance | Agreement/Understanding | The care recipient and the care partner agreed upon how they thought the care recipient would respond to the questions in the LEAD Guide and what they would want in terms of their EOL care; strong shared mutual understanding of care recipient’s values and preferences; Care partner was able to accurately predict care recipient’s responses. | “Both of us are in agreement with my wishes.”  “The more we talked, the more we confirmed that we find ourselves in agreement.”  “We agreed on almost all answers.” |
|  |  | Confidence | One dyad member’s belief that the other will be able to follow the end-of-life wishes outlined during the ACP conversation and or in ACP documents such as an advance directive or the LEAD Guide | "I have a high level of confidence in my ability to make decisions that reflect his end-of-life wishes.”  “Confident that [care partner] will carry out my wishes as discussed an agreed upon.” |
|  |  | Discussion | Conversation about advance care planning; turn-taking by each dyad member; can include clarification achieved through discussion/conversation | “We sometimes needed extra discussion in order to confirm our understanding of what the other person was trying to communicate.”  “We resolved the differences. It was more related to a misunderstanding of the question asked and our post discussion resolved it.” |
| H2: Engaging in ACP is associated with higher interpersonal connectedness. | Engaging in ACP  Fostering higher interpersonal connectedness | Ease of discussion | Conversation in module 3 was easy, no big deal, did not take a lot of effort, was comfortable, etc. | “Conversation was reasonably easy.”  “It was easy and comfortable.”  “It was easy and lovely.” |
|  |  | Transparency | Description of being completely open, honest, and or sharing one’s point of view; not holding back one’s thoughts or feelings | "We had a long discussion about involving a palliative care doctor in my team. He was not aware of how that works, and he was not aware of how important that person was when my best friend died. I feel better knowing that he understands that he would not have to make this decision.” |
|  |  | Unity | Care recipient or care partner describes feeling close to the other participant, that they are in this together, and that they are a good team or are working as a team | “We are "reading each other" more effectively.”  “We have a loving and mutually contributing relationship. We work together well.”  “Our conversation and togetherness becomes more close.” |

Notes. ACP = advance care planning; H1 = hypothesis 1; H2 = hypothesis 2
